# A High Fat/High Sucrose Diet Alters the Skeletal Response to Adenine-Induced Chronic Kidney Disease in Male Rats

**DOI:** 10.1007/s00223-026-01479-w

**Published:** 2026-01-24

**Authors:** Corinne E. Metzger, Landon Y. Tak, Alec N. LaPlant, Matthew R. Allen

**Affiliations:** 1 Department of Anatomy, Cell Biology and Physiology, Indiana University School of Medicine, 635 Barnhill Drive, MS 5045, Indianapolis, IN 46202 USA; 2 Department of Medicine, Division of Nephrology, Indiana University School of Medicine, Indianapolis, IN 46202 USA; 3 Roudebush Veterans Administration Medical Center, Indianapolis, IN 46202 USA

**Keywords:** Chronic kidney disease, Cortical porosity, Parathyroid hormone, High fat/High sucrose

## Abstract

**Background:**

Chronic kidney disease (CKD) impacts a large and growing proportion of the population. Fracture rates are high in individuals with CKD compared to the non-CKD population. Dietary patterns consisting of higher fat and sugar intake are associated with higher risk of developing CKD, but the impact of different dietary patterns on the skeleton in the setting of CKD is largely unknown.

**Objective:**

To assess the impact of a high fat/high sucrose diet (HFHS) in male Sprague Dawley rats with adenine-induced CKD (Ad).

**Methods:**

Rats were given the HFHS or standard diet (SD) for 4 weeks followed by 8 weeks with adenine incorporated into those diets for the Ad groups.

**Results:**

All Ad rats, regardless of diet, had high circulating blood urea nitrogen and parathyroid hormone (PTH). Ad + SD rats had greater femoral volumetric cortical porosity and pore number and lower mechanical properties than Ad + HFHS rats. Ad + HFHS had a lower percentage of cortical bone osteocytes positive for PTHR1 and RANKL matching trends in porosity; however, the HFHS diet led to greater TNF-α-positive osteocytes and trabecular osteoclast numbers.

**Conclusions:**

There were differences in the skeletal response to adenine-induced CKD based on diet with the standard diet leading to a skeletal phenotype more associated with high PTH. These data demonstrate both the complexity of systemic alterations impacting bone in CKD and highlight the importance of understanding the influence of dietary factors on skeletal outcomes.

**Supplementary Information:**

The online version contains supplementary material available at 10.1007/s00223-026-01479-w.

## Introduction

More than one in seven individuals in the United States have chronic kidney disease (CKD) [1]. Fracture risk is high in CKD patients [2–5] and secondary complications of fractures, including increased post-fracture mortality, are elevated in CKD patients compared to the general population [6, 7]. The prevalence of CKD is expected to rise over the next decades, due in part to increasing incidences of diseases linked to CKD development including hypertension and type 2 diabetes [8, 9]. Common dietary patterns may contribute to the increase in CKD and its comorbid conditions. For example, the Western-type diet pattern consisting of high fat, sugar, and processed food intake is associated with a higher risk of CKD [10, 11]. Additionally, a diet high in fried foods and sweetened beverages has been independently associated with mortality in patients with CKD [12]. The impact of dietary patterns, particularly a high fat/high sucrose diet, on the skeletal complications of CKD is largely unknown.

Preclinical models of CKD can recapitulate the high parathyroid hormone (PTH) CKD phenotype which includes high osteoclasts, elevated bone turnover, higher cortical porosity, and lower mechanical properties of bone [13]. In rodent models without CKD, high fat diets decrease bone mass and increase osteoclast activity [14, 15] as well as increase circulating inflammatory factors like TNF-α [16]. It has been proposed that low-grade systemic inflammation may be a link between high fat diets and bone loss [17]. In the context of CKD, the impact of a high fat/high sugar diet on bone outcomes is poorly understood. Therefore, in this current study we aimed to assess the impact of a high fat/high sucrose diet on bone in the adenine-induced CKD model. We hypothesized that a high fat/high sucrose diet would lead to lower cortical bone area and mechanical properties compared to CKD animals fed a standard diet.

## Methods

### Animals

**Fig. 1:**
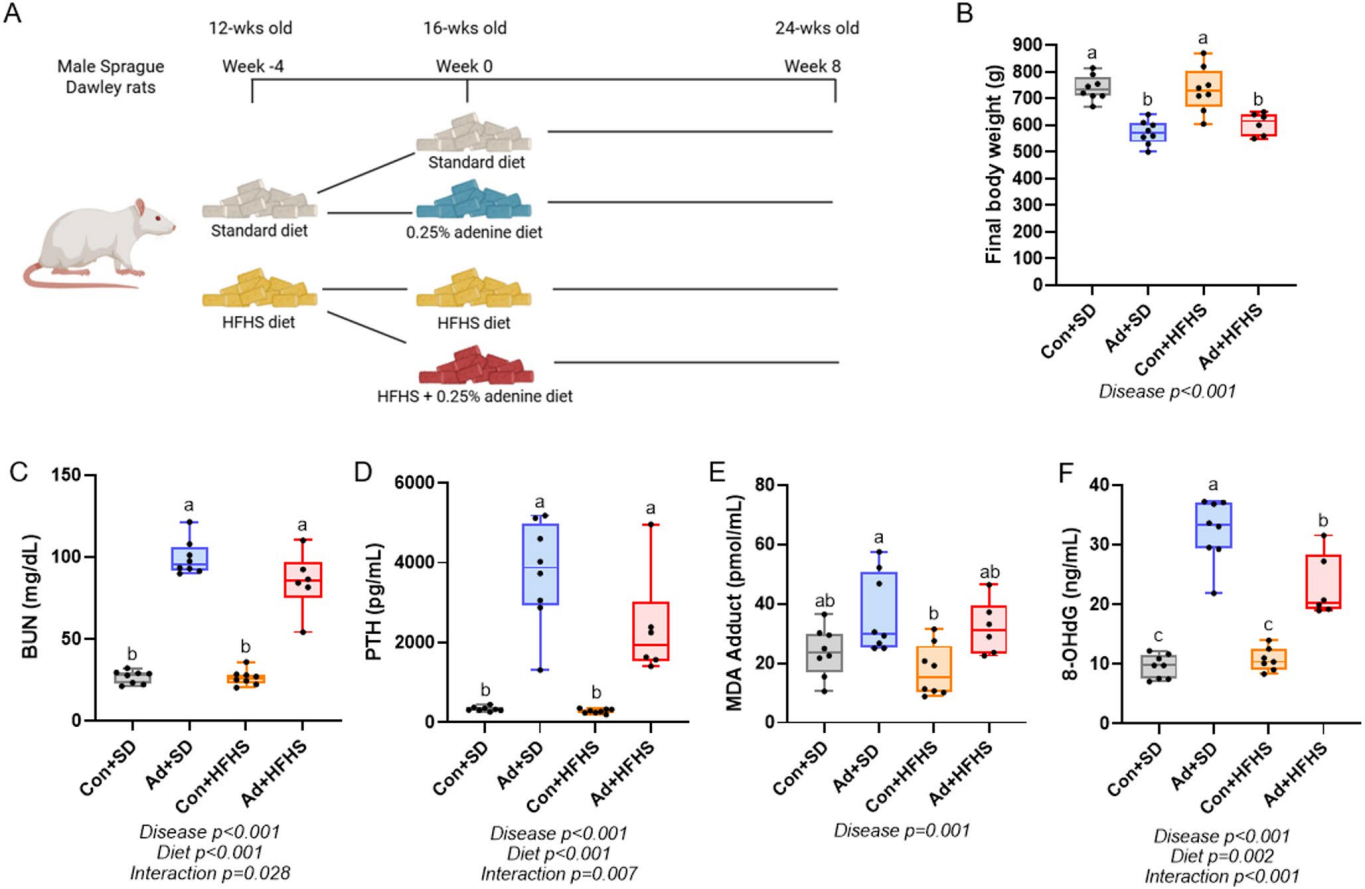
Study design, final body weight, and serum markers. **A** Schematic of the study design. **B** Final body weight was lower in both adenine groups compared to control groups. **C** Serum BUN was higher in both adenine groups compared to both control groups. **D** PTH was higher in both adenine groups compared to both control groups with no statistical effect of diet. **E** MDA adduct, a marker of lipid peroxidation, was statistically higher in Ad + SD compared to Con + HFHS. **F** Serum 8-OHdG, a marker of oxidative damage, was higher in both adenine groups compared to control groups with Ad + SD higher than Ad + HFHS. Bars not sharing the same letter are statistically different. Statistically significant disease-by-diet interaction effect noted below the graph when present

 Male Sprague Dawley rats (*n* = 32) were ordered from Inotiv (Madison, WI, USA) at 11 weeks of age and group-housed two per cage at an institutionally approved animal facility with 12-hour light/dark cycles. At 12 weeks of age, half of the rats (*n* = 16) were randomly switched to the standard diet (SD) consisting of a purified casein-based diet with adjusted calcium and phosphorous (0.6% calcium, 0.9% phosphorous; Teklad Diets [TD.150303], Inotiv). The other half of the rats (*n* = 16) were placed on a high fat/high sucrose diet (HFHS) with 42% of Kcal from fat, high sucrose, and the same altered calcium and phosphorus ratio (Teklad Diets [TD.230094], Inotiv). After a four-week diet induction, at 16 weeks of age, half of each group was randomly placed on the same diet formulation with the inclusion of 0.25% adenine to induce chronic kidney disease - Ad + SD (Teklad Diets [TD.230093]) and Ad + HFHS (Teklad Diets [TD.230095]) (Table 1). The groups remained on their specific diets for eight weeks (Fig. 1A). Casein serves as the protein base for many rodent purified diets including those formulated by the American Institute of Nutrition and widely utilized in studies of all types (rodent diets AIN-93G and AIN-93 M) and has been utilized as the protein base by our group in various different CKD models [13, 18–20]. Body weight was measured weekly throughout the study and all rats maintained a normal body condition. Two rats in the Ad + HFHS group were euthanized at study weeks 7 (23 weeks of age) and 8 (24 weeks of age) due to the sudden development of hindlimb paralysis. Necropsy did not identify a clear cause. Both rats were excluded from analyses leaving the Ad + HFHS group with final *n* = 6. After 8 weeks on the adenine diets (24 weeks of age), animals were anesthetized via vaporized inhaled isoflurane and euthanized via exsanguination and thoracotomy. All animal procedures were approved by the Indiana University School of Medicine Animal Use and Care Committee (protocol #21175) prior to the initiation of experimental protocols and methods were carried out in accordance with relevant guidelines and regulations.

**Table 1:** Dietary components of the standard diet, standard adenine diet, high fat/high sucrose (HFHS), and HFHS adenine diet

Component	Standard	Standard Adenine	HFHS	HFHS Adenine
Protein (% of total kcal)	19.9	20	15.3	15.3
Carbohydrate (% of total kcal)	66.9	66.8	42.5	42.4
Fat (% of total kcal)	13.2	13.2	42.2	42.3
Total Kcal/g	3.5	3.5	4.5	4.5
Casein (g/Kg)	200	200	195	195
DL-Methionine (g/Kg)	3	3	3	3
Corn Starch (g/Kg)	397.93	395.33	150	150
Maltodextrin (g/Kg)	140	140	0	0
Sucrose (g/Kg)	92.3	92.3	341.06	338.56
Corn Oil (g/Kg)	50	50	0	0
Cellulose (g/Kg)	50	50	39.73	39.73
Calcium Phosphate, dibasic, dihydrate (g/Kg)	20	20	0	0
Calcium Phosphate, monobasic, monohydrate (g/Kg)	0	0	31.2	31.2
Sodium Phosphate, monobasic, monohydrate (g/Kg)	12	12	0	0
Sodium Phosphate, dibasic (g/Kg)	6	6	0	0
Calcium Carbonate (g/Kg)	3.4	3.4	5	5
Choline Bitartrate (g/Kg)	2	2	0	0
Adenine (g/Kg)	0	2.5	0	2.5
Ethoxyquin, antioxidant (g/Kg)	0	0	0.04	0.04
Cholesterol (g/Kg)	0	0	1.5	1.5
Anhydrous Milkfat (g/Kg)	0	0	210	210

### Serum Markers

 Serum from cardiac blood was used to assess blood urea nitrogen (BUN) via colorimetric assay (BioAssay Systems, Hayward, CA, USA). Serum intact parathyroid hormone (Immnotopics Quidel, San Diego, CA, USA), malondialdehyde (MDA) adduct (Abcam, Cambridge, MA, USA), and 8-hydroxy-2’-deguanosine (8-OHdG; Enzo Life Science Inc., Farmingdale, NY, USA) were assessed via ELISA. All samples were assayed in duplicate following manufacturer protocols.

### Micro-Computed Tomography

 The NBF-fixed right proximal tibia metaphysis and the PBS-soaked frozen right femoral midshaft were scanned on a SkyScan 1272 (Bruker, Billerica, MA, USA) with a 0.5 mm aluminum filter and a 12-micron voxel size. For the proximal tibia, trabecular bone was analyzed in a 1 mm region beginning approximately 0.5 mm distal to the proximal growth plate. For the femoral midshaft, cortical bone parameters were obtained in a 1 mm section with a region of interest hand-traced on the periosteal and endosteal surfaces. Cortical bone area was obtained using a binary threshold of 110 to 255. Cortical thickness was obtained with a flooded binary threshold (0 to 255) to account for the width of the endosteal to periosteal surfaces without porosity. The cortical pore volumetric network was analyzed with a reverse binary threshold of 110 to 0 to quantify percent volumetric cortical porosity, average pore length, average distance between pores, pore number, and connectivity density within the pore network.

### Mechanical Testing

 At the time of mechanical testing, the same femora that were micro-CT scanned were thawed at room temperature (from storage in saline-soaked gauze at -20 °C) and remain soaked in saline until mechanical testing to maintain hydration of samples. Anterior-posterior (AP) diameter was obtained using calipers at the femoral midshaft. Bones were tested to failure using a mechanical testing system (Test Resources, Shakopee, MN, USA) in 3-point bending (lower support span 18 mm). A 667 N load cell and a 2 mm/min displacement rate were used for all tests. Bones were oriented with the anterior surface of the femur in compression with the loading point at 50% bone length. All bones were pre-loaded with ~ 0.5 N. Mean polar moment of inertia was obtained from micro-CT analyses and diameters from calipers were used to estimate material level properties using standardized bending equations. A custom MATLAB script was used to determine structural and estimated material level properties.

### Histomorphometry

 Following micro-CT, the fixed right proximal tibiae were serially dehydrated through graded steps of ethanol and subsequently embedded in methyl methacrylate (Sigma Aldrich, St. Louis, MO). Serial frontal sections were cut 4 μm thick and stained with Von Kossa/McNeal stain for assessment of osteoclast-covered trabecular surfaces (Oc.S/BS, %) osteoclast number (Oc.N/B.Pm, #/mm), trabecular osteoid surfaces (OS/BS, %), and osteoid width (OW, mm). A standard region of interest of trabecular bone excluding primary spongiosa and endocortical surfaces near was analyzed. All analyses were performed using BIOQUANT (BIOQUANT Image Analysis, Nashville, TN). All nomenclature for histomorphometry follows standard usage [21].

### Immunohistochemistry

A section of fixed right tibia midshaft was decalcified in 14% EDTA and embedded in paraffin. Serial 5 μm sections were taken. Sections were stained utilizing a standard avidin-biotin method (Vector Laboratories, Burlingame, CA, USA). Samples were stained for the parathyroid hormone receptor-1 (PTHR1; Abcam #ab75150), tumor necrosis factor-α (TNF-α; Abcam #ab34674), and receptor activator of nuclear factor κB ligand (RANKL; Abcam #ab62516) with rabbit polyclonal antibodies. For 8-OHdG, a mouse monoclonal antibody was used (Abcam #ab48508). Peroxidase development was performed with an enzyme substrate kit (DAB, Vector Laboratories). Counterstaining was conducted with methyl green (Vector Laboratories). Negative controls for all antibodies were completed by omitting the primary antibody. Sections were analyzed as the percentage of osteocytes stained positively for the protein (DAB-positive) relative to all osteocytes (DAB-positive and methyl green-positive) in the cross-section. All analyses were completed by the same individual.

### Statistical Analyses

 Data were assessed for normality using Shapiro-Wilk. If data were normally distributed, data were analyzed with a 2 × 2 Factorial ANOVA (disease-by-diet) with all main and interaction effects recorded. When the model 2 × 2 ANOVA p-value was statistically significant, a Tukey HSD post hoc test was completed to assess individual group differences. When data did not meet normality assumptions, data were processed via align-and-rank procedure for nonparametric multifactorial ANOVA utilizing the ARTool [22] followed by an ANOVA assessment for each factor as defined by the ART protocol. All main and interaction effects were recorded. Pairwise comparisons following the ART ANOVA were completed with an aligned rank transform procedure for multifactor contrasts (ART-C) [23]. Following the ART procedure for contrasts, post hoc analyses were run with a Tukey HSD. All data are presented as mean ± standard deviation. Volumetric pore data (between Ad + SD and Ad + HFHS) were completed with a t-test or a Wilcoxon test for non-parametric data. Linear regression analyses were performed within dietary treatment (standard diet and HFHS diet) with serum PTH as the independent factor and osteocyte PTHR1 and RANKL separately as dependent variables. All statistical analyses were completed on JMP Pro 17 (SAS, Cary, NC, USA).

## Results

### Body Weights Showed an Effect of disease, but not Diet

At baseline, there were no differences in body weight between groups (*p* = 0.552). After four weeks of either the standard or HFHS diet, there were also no differences between groups (*p* = 0.076; Suppl Fig). At the study endpoint, there was a main effect of disease (*p* < 0.001), but no effect of diet (*p* = 0.537) and no diet-by-disease interaction effect (*p* = 0.368). Both adenine groups weighed less than animals in both control groups (Fig. 1b). Overall, in the 8-week adenine period, Ad groups maintained weight, while Con groups gained weight (Suppl Fig).

### Serum BUN and PTH Were Higher in Adenine-Induced CKD in Both Diets

BUN (ART ANOVA) showed a main effect of disease (*p* < 0.001) and diet (*p* < 0.001), and an interaction (*p* = 0.028). Both adenine groups had higher BUN than both control groups (Fig. 1C). PTH (ART ANOVA) showed main effects of disease (*p* < 0.001), diet (*p* < 0.001), and an interaction (*p* = 0.007) with both adenine groups having higher circulating levels of PTH compared to both control groups (Fig. 1D).

### Serum Markers of Oxidative Stress Were Higher in Adenine-Induced CKD. Ad + HFHS had Lower 8-OHdG than Ad + SD

Serum MDA adduct (2 × 2 ANOVA), a marker of lipid peroxidation and oxidative stress, showed a main effect of disease (*p* = 0.001), but no effect of diet (*p* = 0.147) and no diet-by-disease interaction (*p* = 0.816). The Ad + SD group had higher levels of MDA adduct compared to the Con + HFHS group (Fig. 1E). Serum 8-OHdG (ART ANOVA), a marker of DNA damage from oxidative stress, had effects of disease (*p* < 0.001), diet (*p* = 0.002), and an interaction effect (*p* < 0.001). Ad + SD had higher serum 8-OHdG compared to Ad + HFHS, while both control groups had the lowest values (Fig. 1F).

### Cortical Porosity was Present in Both Adenine Groups at the Midshaft Femur with no Differences in Cortical Thickness

**Fig. 2:**
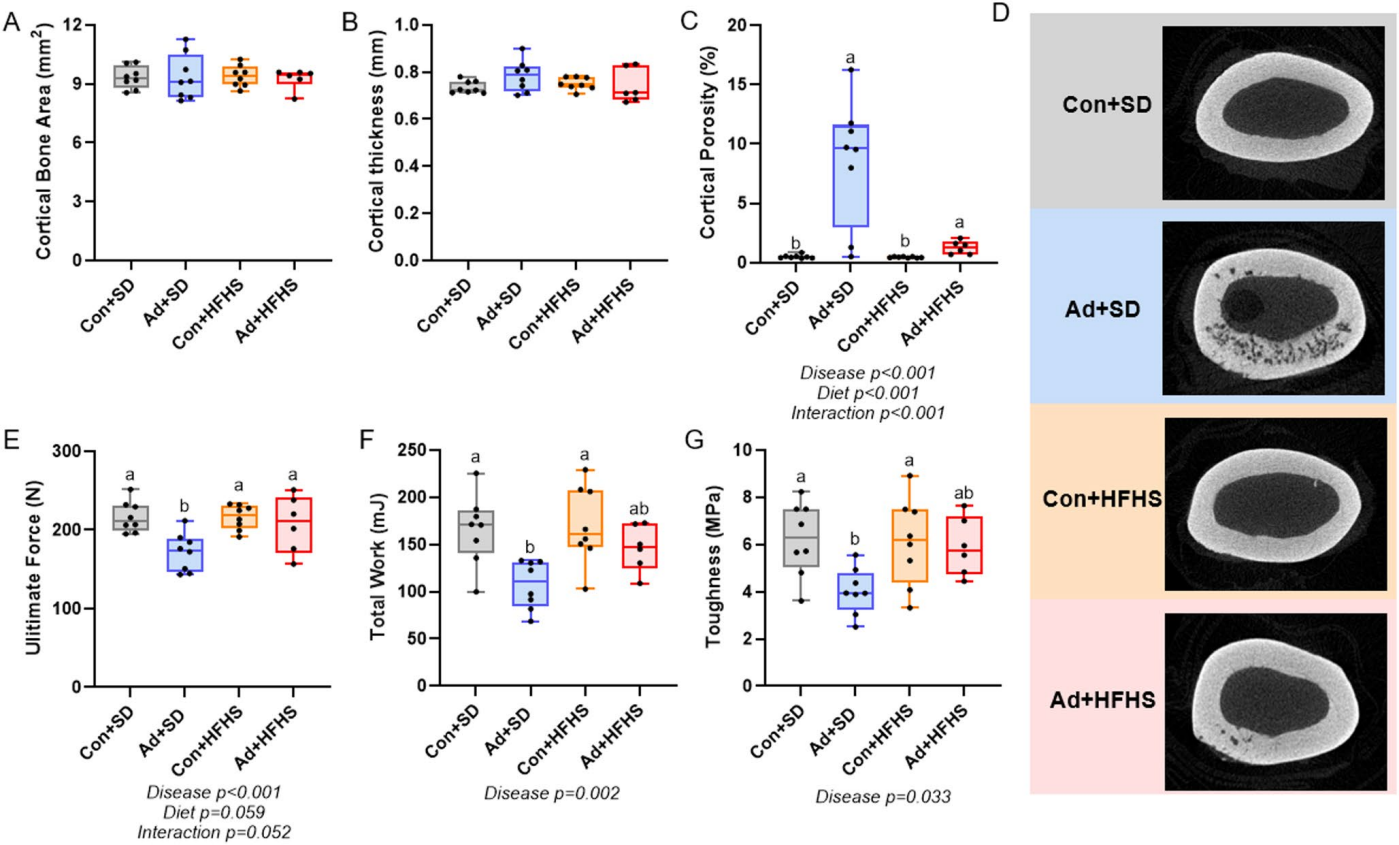
Midshaft femur micro-CT and mechanical properties of bone. **A** Cortical bone area was not different between groups. **B** There were no differences between groups in cortical thickness. **C** Cortical porosity was higher in both adenine groups compared to both control groups. **D** Representative images of the femur midshaft from micro-CT. Images represent the sample with cortical porosity closest to the group mean. **E** Ultimate force was lower in Ad + SD compared to all other groups. **F** Total work was lower in Ad + SD compared to both control groups. **G** Toughness was lower in Ad + SD compared to both control groups. Ad + HFHS was not different from any group. Bars not sharing the same letter are statistically different. Statistically significant disease-by-diet interaction effect noted below the graph when present

At the midshaft femur, there were no differences in total bone area (*p* = 0.377), cortical bone area (*p* = 0.991; Fig. 2A), or cortical thickness (*p* = 0.266; Fig. 2B). Cortical porosity (ART ANOVA) had main effects for disease (*p* < 0.001), diet (*p* < 0.001), and an interaction (*p* < 0.001) with both adenine groups higher than both control groups (Fig. 2C). Representative images of the femoral midshaft of the sample closest to the mean for cortical porosity are in Fig. 2D.

### Ad + SD had Lower Ultimate Force than all other Groups

There was a main effect of disease (*p* = 0.005) for ultimate force, but no statistical effect of diet (*p* = 0.059) or diet-by-disease (*p* = 0.052). Ad + SD rats had lower ultimate force than all other groups (Fig. 2E). There was a main effect of disease (*p* = 0.002) for total work, but no effect of diet (*p* = 0.083) or an interaction effect (*p* = 0.177). Ad + SD rats had lower total work than both control groups, but were not statistically different from Ad + HFHS rats (Fig. 2F). There were no statistical differences in stiffness (*p* = 0.094). There was a main effect of disease (*p* = 0.033) for toughness, but no effect of diet (*p* = 0.114) or an interaction effect (*p* = 0.072). Ad + SD rats had lower toughness than all other groups (Fig. 2G). The statistical model did not show significant differences modulus (*p* = 0.227), post-yield displacement (*p* = 0.215), or total displacement (*p* = 0.087; Table 2).

**Table 2:** Mechanical properties from femur 3-pt-bend testing

	Con + SD	Ad + SD	Con + HFHS	Ad + HFHS
Stiffness (N/mm)	491 ± 102	398 ± 53	455 ± 69	404 ± 887
Postyield Displacement (µm)	709 ± 168	577 ± 92	723 ± 205	598 ± 172
Total Displacement (µm)	1061 ± 178	900.2 ± 105	1113 ± 215	1025 ± 124
Modulus (MPa)	3.75 ± 0.60	3.20 ± 0.85	3.59 ± 0.48	3.08 ± 0.78

Data are reported as mean ± standard deviation. Groups not sharing the same letter are statistically different

### Ad + HFHS had Lower Cortical Pore Number and Lower Pore Connectivity Within the Midshaft Femur than Ad + SD

**Fig. 3:**
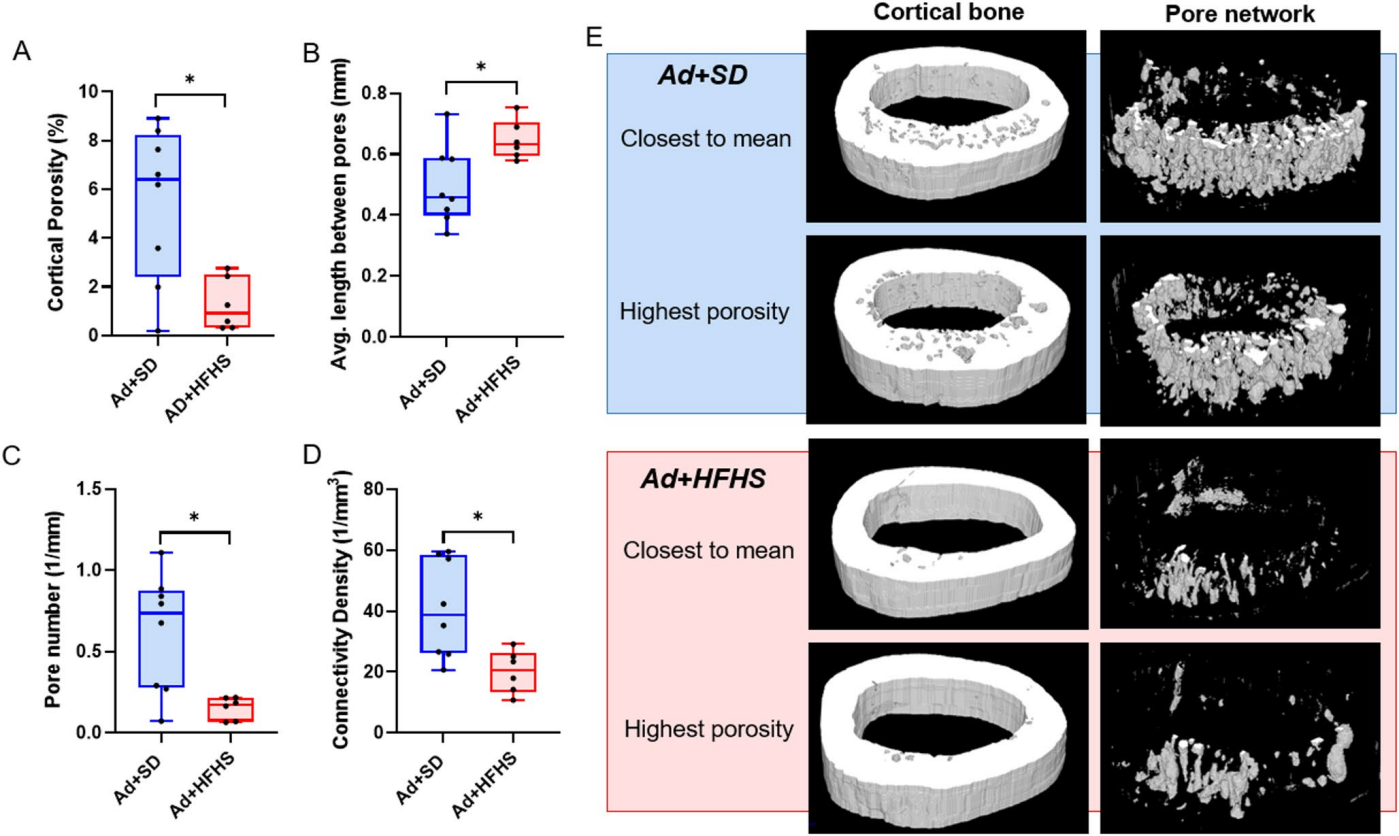
Volumetric pore network analysis of the midshaft femur in adenine groups. **A** Volumetric cortical porosity was lower in Ad + HFHS than Ad + SD. **B** Average length between pores was higher in Ad + HFHS. **C** Pore number was lower in Ad + HFHS vs. Ad + SD. **D** Connectivity density of the pore network was lower in Ad + HFHS. *Indicates difference in t-test between the two groups. **E** Representative images of cortical bone with a 100–255 binary threshold (left) and the inverse threshold (100-0) to visualize the pore network in the samples closest to the mean and highest for volumetric cortical porosity

From the 1 mm regions of interest at the midshaft femur, Ad + SD had higher cortical porosity than did Ad + HFHS (Mann-Whitney U *p* = 0.043; Fig. 3A). Average pore length was not different between groups (*p* = 0.576). Average length between pores was greater in Ad + HFHS (*p* = 0.023; Fig. 3B) and pore number was higher in Ad + SD (Mann-Whitney U *p* = 0.008; Fig. 2C). Pore connectivity density was also higher in the Ad + SD group vs. Ad + HFHS group (Mann-Whitney U *p* = 0.013; Fig. 3D). Representative images of the pore network are presented in Fig. 2E.

### Tibial Trabecular Bone Microarchitecture Showed Minimal Diet or Disease Effects

At the proximal tibia, there was no difference in trabecular bone volume/total volume (BV/TV) across the groups (*p* = 0.097). Other trabecular variables are reported in the Supplemental Figure.

### Both Adenine Groups had Higher Osteoclast-Covered Trabecular Surfaces than Control rats, but Ad + HFHS had Higher Osteoclast Number than Ad + SD

**Fig. 4:**
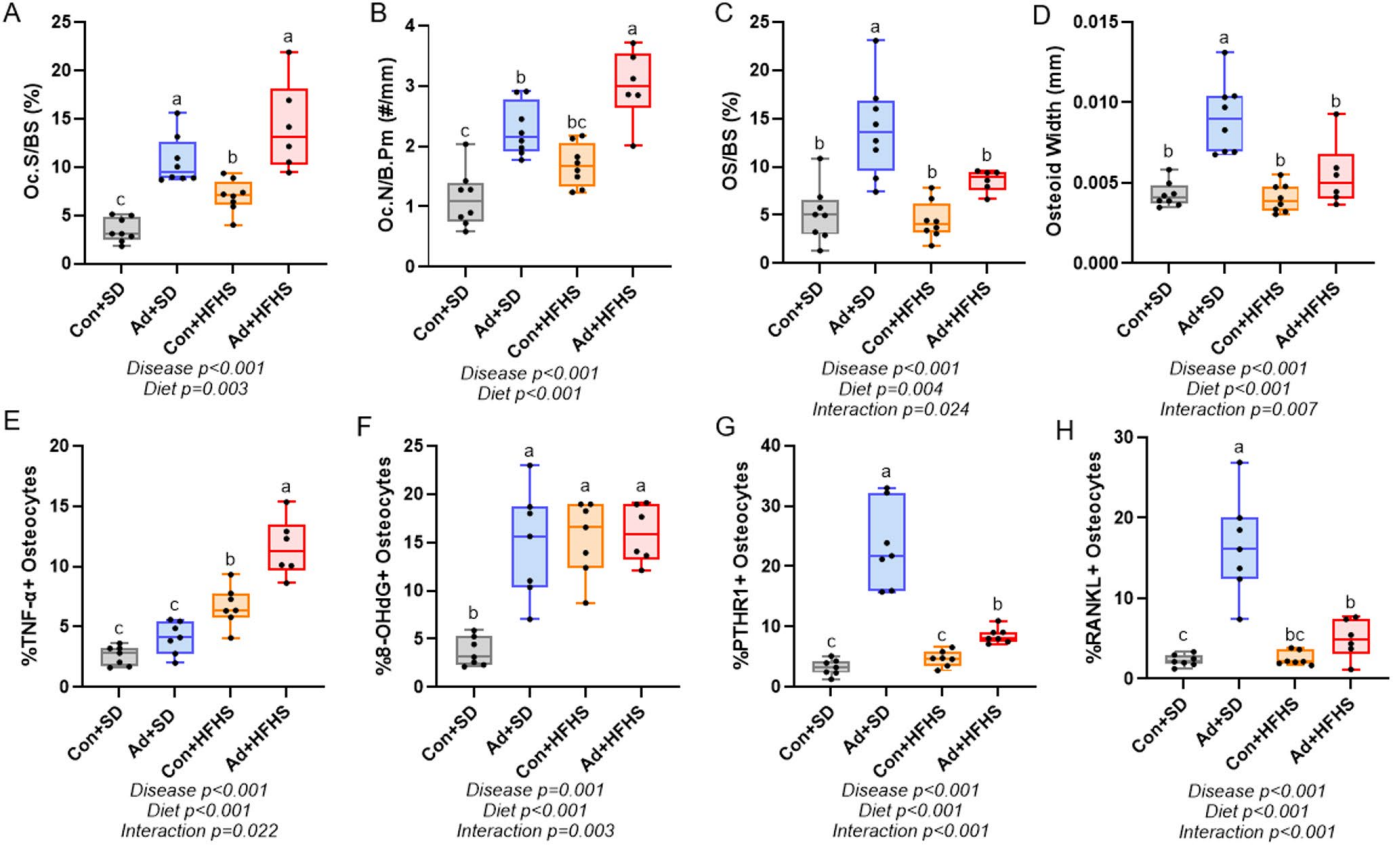
Static histomorphometry of the proximal tibia metaphysis (A-D) and immunohistochemical analyses of cortical osteocytes in the midshaft tibia (E-H). **A** Osteoclast-covered trabecular surfaces were higher in both adenine groups compared to both control groups. **B** Osteoclast numbers were highest in Ad + HFHS followed by Ad + SD with Con + SD having the lowest group average. **C** Osteoid-covered trabecular surfaces were higher in Ad + SD compared to all other groups. **D** Osteoid thickness was highest in Ad + SD with no differences between the other groups. **E** %TNF-α-positive osteocytes were highest in Ad + HFHS followed by Con + HFHS with Con + SD having the lowest group average. **F **Con + SD had lower %8-OHdG-positive osteocytes compared to all other groups. **G** %PTHR1-positive osteocytes were highest in Ad + SD followed by Ad + HFHS with both control groups having the lowest values. **H** Ad + SD had higher %RANKL-positive osteocytes compared to all other groups. Bars not sharing the same letter are statistically different. Statistically significant disease-by-diet interaction effect noted below the graph when present

Oc.S/BS (ART ANOVA) showed a main effect of both disease (*p* < 0.001) and diet (*p* = 0.003), but no interaction effect (*p* = 0.841). Both adenine groups had greater Oc.S/BS compared to both control groups (Fig. 4A). Oc.N/B.Pm showed a main effect of both disease (*p* < 0.001) and diet (*p* < 0.001), but no interaction effect (*p* = 0.610). Ad + HFHS had the highest value for osteoclast numbers followed by Ad + SD with the Con + SD group having the lowest values (Fig. 4B).

### Osteoid-Covered Trabecular Surfaces and Osteoid Width were Higher in Ad + SD Compared to Ad + HFHS

OS/BS (ART ANOVA) had main effects of disease (*p* < 0.001) and diet (*p* = 0.004) and an interaction effect (*p* = 0.024) Ad + SD mice had greater OS/BS compared to all other groups (Fig. 4C). Osteoid width (ART ANOVA) showed effects of disease and diet (*p* < 0.001 for both) and an interaction (*p* = 0.007). Ad + SD rats also had greater osteoid width than all other groups (Fig. 4D).

### %TNF-α- and 8-OHdG-Positive Osteocytes were Elevated in the HFHS Diet Groups

%TNF-α+-osteocytes (ART ANOVA) had main effects of disease and diet (*p* < 0.001 for both) and an interaction effect (*p* = 0.022) with AD + HFHS rats having the highest group values followed by Con + HFHS and then both standard diet groups (Fig. 4E). %8-OHdG-+-osteocytes (ART ANOVA) also showed main effects of disease (*p* = 0.001), diet (*p* < 0.001), and an interaction (*p* = 0.003) with Con + SD lower than all other groups (Fig. 4F).

### Both %PTH1R-Positive- and %RANKL-Positive- Osteocytes Were Elevated in Ad + SD, but not Similarly Impacted in the Ad + HFHS Group

Both %PTH1R-+-osteocytes (ART ANOVA) and %RANKL-+-osteocytes (ART ANOVA) showed all main and interaction effects (*p* < 0.001 for all). Ad + SD rats had the highest %PTH1R-+-osteocytes followed by Ad + HFHS and then both control groups (Fig. 4G). %RANKL-+-osteocytes were highest in Ad + SD compared to all other groups (Fig. 4H).

### Serum PTH Statistically Predicted %PTHR1 + Osteocytes in the Standard Diet Groups, but not in HFHS Diet Groups

**Fig. 5:**
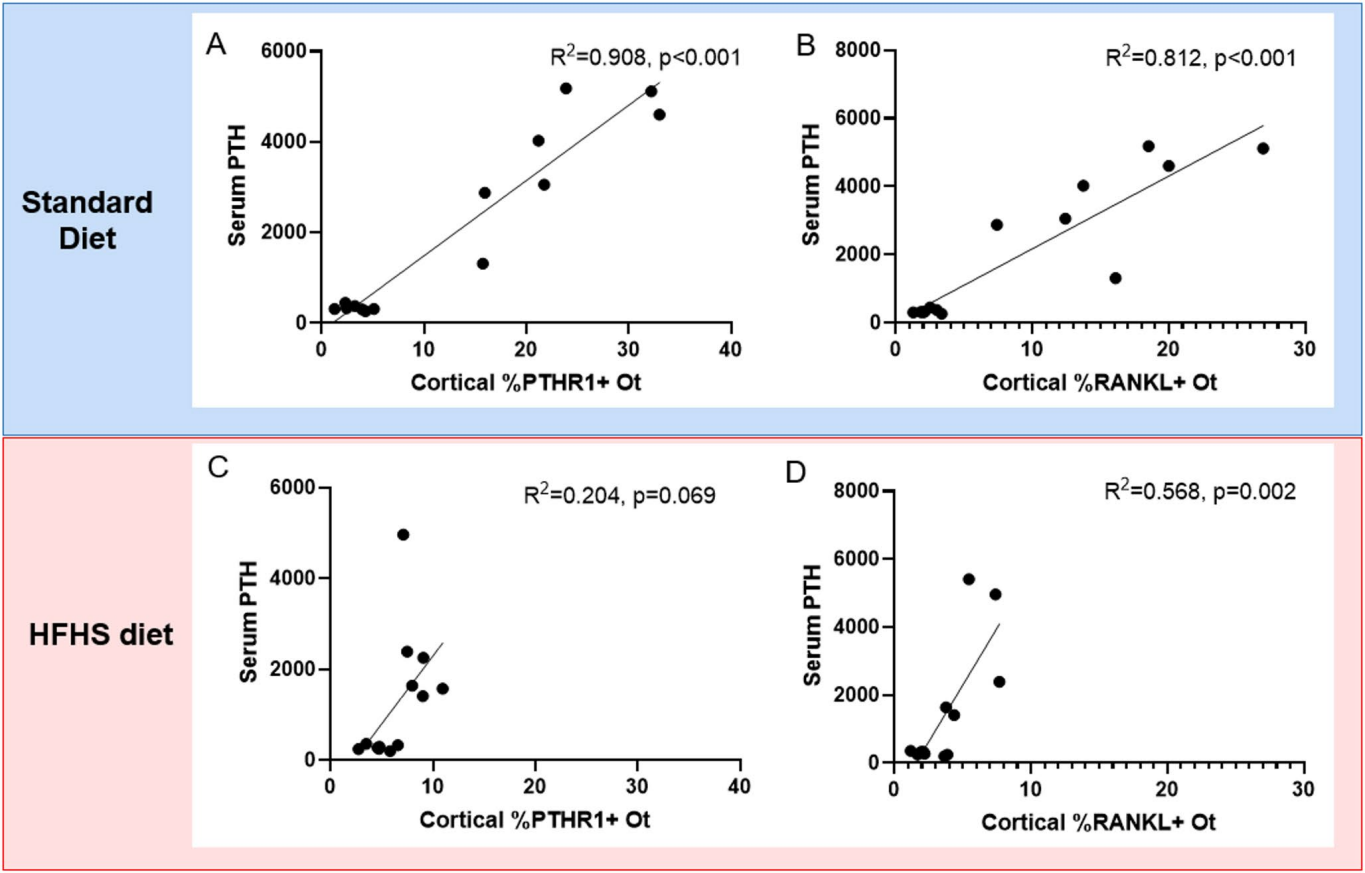
Regression analyses between serum PTH and %PTHR1 + and %RANKL + osteocytes. **A** Serum PTH was statistically related to cortical %PTHR1 + osteocytes (R^2^ = 0.908, *p* < 0.001) in standard diet control and adenine-induced CKD groups. **B** Within standard diet groups, serum PTH was statistically predictive of %RANKL + osteocytes (R^2^ = 0.812, *p* < 0.001). **C** There was no statistical relationship between serum PTH and cortical %PTHR1 + osteocytes in HFHS diet groups (R^2^ = 0.204, *p* = 0.069). **C** Serum PTH was statistically related to %RANKL + osteocytes in HFHS diet groups (R^2^ = 0.568, *p* = 0.0021)

Within the standard diet groups, serum PTH was statistically predictive of %PTHR1 + osteocytes via regression analysis (R^2^ = 0.908, *p* < 0.001; Fig. 5A). Similarly, serum PTH statistically predicted the variability in %RANKL + osteocytes within the standard diet groups (R^2^ = 0.812, *p* < 0.001; Fig. 5B). Within the HFHS diet groups, there was no statistical relationship between serum PTH and %PTHR1 + osteocytes (R^2^ = 0.204, *p* = 0.069; Fig. 5C). %RANKL + osteocytes were statistically predicted by serum PTH within the HFHS diet groups (R^2^ = 0.568, *p* = 0.002; Fig. 5D).

## Discussion

The primary finding of this study is that adenine-induced CKD had diet-dependent skeletal effects. Contrary to our original hypothesis, a high fat/high sucrose diet did not lead to greater cortical bone deterioration. Our data alludes to the high fat/high sucrose diet altering the skeletal response to CKD, specifically to PTH, resulting in more modest cortical structural changes. Despite lower cortical bone changes, the bone inflammatory state and trabecular osteoclast numbers were highest in CKD with high fat/high sucrose. This highlights the complexity of systemic interactions influencing skeletal changes in CKD.

High fat diets have been linked with higher oxidative stress [24, 25]; likewise, CKD is associated with increased oxidative stress in human [26, 27] and animal studies [19, 28]. In our study, the two systemic markers of oxidative stress assessed primarily showed CKD-induced differences with higher values in adenine-induced CKD groups; however, circulating 8-hydroxy-2′-deoxyguanosine (8-OHdG), a marker of oxidative DNA damage, was statistically different (32% lower) in the high fat/high sucrose adenine group compared to the standard adenine group. While all adenine rats had decreased kidney function measured by high serum BUN, dietary factors may have contributed to modest differences in oxidative stress. Interestingly, osteocyte 8-OHdG in cortical bone was elevated due to adenine-induced CKD only in the standard diet as both high fat/high sucrose diet groups had higher 8-OHdG regardless of CKD status. These data differ from the systemic serum markers of oxidative stress indicating a potential systemic vs. local tissue difference in oxidative stress with greater bone/osteocyte oxidative stress from diet alone.

Bone loss in CKD particularly targets cortical bone. This cortical bone loss can occur rapidly in both patients with CKD [29] and preclinical rodent models of CKD [18, 30]. Particularly in preclinical models, the increase in porosity is associated with higher circulating PTH [18, 20]. In our current study, analyses of the pore network at the midshaft femur showed greater overall porosity and pore number in the standard diet adenine group compared to the HFHS diet group. These differences translated to a mechanical effect, where the standard diet adenine group had 20% lower ultimate load compared to control groups while the high fat/high sucrose diet adenine group was not statistically different from controls (5% lower than control groups). The higher number of pores and greater connectivity of the pore network in the standard diet group is likely what drove the differences in the mechanical properties as there were no differences in cortical bone area or thickness. Serum PTH in our model was not statistically different between both adenine-CKD groups, although there was a main effect of diet with the HFHS adenine-CKD group having numerically lower values than the standard diet group. This PTH effect likely contributes to the significantly lower cortical porosity in the HFHS diet group; however, how diet impacts circulating PTH is unknown.

Generally, continuously high PTH triggers bone loss through increased osteoclastic drive which leads to high bone turnover. Preclinical models of CKD show that high PTH is associated with higher osteoclast-covered surfaces/osteoclast numbers as well as high bone formation rate [13, 31]. Independent of PTH, pro-inflammatory cytokines are associated with increased osteoclasts [32, 33]. In our current study, we found that both adenine groups had higher osteoclast-covered trabecular surfaces compared to both control groups. Additionally, trabecular osteoclast numbers were higher in the high fat/high sucrose adenine group compared to the standard adenine group. Although not statistically different, the high fat/high sucrose adenine-CKD had 39% lower trabecular bone volume compared to the diet-matched control which matches the osteoclast number data. The lower cortical porosity in the high fat/high sucrose adenine group suggests differences in osteoclast activity between trabecular bone and intracortical bone within the diet groups. While we could not directly assess bone formation rate in this study, we did find higher osteoid-covered trabecular surfaces and osteoid thickness in the standard diet adenine rats indicating a high turnover state; however, the high fat/high sucrose diet adenine group did not have similar increases in osteoid. Together these data suggest a pro-resorptive state in the high fat/high sucrose CKD group that does not correspond with a high osteoid bone phenotype in CKD. Interestingly, despite the differing responses in bone turnover, there were no differences in trabecular bone volume across all the groups including the control groups. More notable differences may appear with longer duration of disease and diet.

To further explore these differences due to diet, we histologically assessed PTH receptor 1 (PTHR1) in cortical osteocytes. Adenine-induced CKD on the standard diet had 8-fold higher %PTHR1 + osteocytes than standard diet controls. In contrast, adenine-induced CKD on the high fat/high sucrose diet had only 2-fold higher %PTHR1 + osteocytes than diet-matched controls. Following a similar pattern, %RANKL + osteocytes were 7-fold higher in standard diet adenine rats vs. control standard diet rats while high fat/high sucrose diet adenine rats only had 2-fold higher %RANKL + cortical osteocytes than diet-matched controls. In healthy mice, constitutively active PTHR1 in osteocytes led to elevations in RANKL and cortical bone alterations including intracortical remodeling and cortical porosity [34]. Previously, we showed that osteoblast/osteocyte-derived RANKL is critical for the development of cortical porosity and high bone turnover in adenine-induced CKD mice despite high circulating PTH [31]. In our current study, the high fat/high sucrose adenine-induced CKD group had statistically lower cortical osteocytes positive for PTHR1 and RANKL which likely contributed to the less severe cortical phenotype. Linear regression analyses showed that circulating PTH statistically predicted ~ 90% of the variability in osteocyte PTHR1 in the standard diet rats, but there was no statistical relationship between serum PTH and osteocyte PTHR1 in the HFHS diet rats. Similarly, serum PTH was more strongly associated with osteocyte RANKL in standard diet rats (R^2^ = 0.812) vs. HFHS diet rats (R^2^ = 0.568). It is possible that dietary differences could have impacted the timeline of development of CKD-induced cortical bone changes. It is also possible that the high fat/high sucrose diet led to a type of skeletal hyporesponsiveness to PTH. Whether different dietary patterns lead to skeletal hyporesponsiveness to PTH warrants further investigation. Regardless of the cause, in the current study differences in dietary composition in the same treatment period led to differing skeletal responses to CKD.

In cortical bone, we also found differing effects of diet on pro-inflammatory status. Osteocytes positive for TNF-α, a pro-inflammatory cytokine, were ~ 3-fold higher in the high fat/high sucrose diet and even higher in the adenine HFHS group. High fat diet composition is associated with low grade inflammation [17] and previous rodent studies have found higher TNF-α in various tissues due to a high fat diet [35, 36]. Interestingly in our study, osteocytes positive for RANKL were not elevated in the control high fat/high sucrose group compared to diet-matched control despite higher TNF-α. It appears that PTH/PTHR1 was the primary driver of higher RANKL in this model rather than TNF-α.

Limitations of this current study include not being able to assess the impact of obesity on bone parameters in CKD as the HFHS diet rats in our current study did not have higher body weight. Therefore, it is important to note that the results of this study look at the effect of different diet compositions and not obesity. We were unable to obtain accurate food intake measures throughout the study as rats were housed two per cage to minimize stress and individual food intake measures were not possible. Additionally, it is important to note that two rats in the high fat/high sucrose adenine-induced CKD group spontaneously developed hindlimb paralysis in the last 7 days of the study. We were unable to determine the cause of this, but it only occurred within high fat/high sucrose adenine group. Additionally, other studies from our group with adenine-induced CKD rats on the standard diet have gone weeks further without developing similar issues. Therefore, we hypothesize this effect was likely due to the combination of diet and disease state, but the cause remains unclear. We were also unable to obtain detailed measures of kidney function to determine timeline of development of CKD and the impact of diet. Finally, there may be specific interactions between the adenine model of inducing CKD and the diets given here. In the adenine-induced CKD model, functional kidney decline is caused by formation of 2,8-dihydroxyadenine crystal deposits in the renal tubules [37] and how dietary differences impacts this process is unknown.

In conclusion, rats on a high fat/high sucrose diet had differing skeletal responses to adenine-induced CKD most notably with lower cortical porosity and preserved mechanical properties of bone compared to adenine-induced CKD rats on a standard diet. The high fat/high sucrose adenine group had a lower percentage of cortical osteocytes positive for PTHR1 and RANKL compared to the standard diet group which likely contributed to the cortical bone changes and preserved mechanical properties. Despite less PTH-driven skeletal changes, the high fat/high sucrose adenine-induced CKD group had a pro-inflammatory state in bone and high osteoclasts indicating a combined effect of diet and CKD on these parameters. Many preclinical studies use a standard diet for all animals in the studies, therefore, not showing differences due to dietary composition. Further, many clinical studies are unable to tightly control dietary intake which may contribute to the wide variability in renal osteodystrophy responses across patients. Overall, these preclinical data demonstrate dietary composition alters parameters of CKD-induced skeletal alterations.

## Supplementary Information

Below is the link to the electronic supplementary material.

Supplementary Material 1

Supplementary Material 2

## Data Availability

Data described in the manuscript will be made available upon request.
