# Supplementary material for: A High Fat/High Sucrose Diet Alters the Skeletal Response to Adenine-Induced Chronic Kidney Disease in Male Rats
Source: Calcif Tissue Int. 2026 Jan 24;117(1):15. doi: 10.1007/s00223-026-01479-w (PMC12831797; doi:10.1007/s00223-026-01479-w)
Supplement: Supplementary file 1 — Supplementary Material 1 [file 223_2026_1479_MOESM1_ESM.docx]

**Supplemental Figure:** Body weight across the 8 weeks of adenine induction.

**Supplemental Table**: Proximal tibia trabecular bone parameters from micro-CT. Groups not sharing the same letter are statistically different from each other.

|  | **Con+SD** | **Ad+SD** | **Con+HFHS** | **Ad+HFHS** |
| --- | --- | --- | --- | --- |
| BV/TV (%) | 14.88 ± 4.31 | 15.71 ± 3.96 | 16.25 ± 3.79 | 10.53 ± 3.04 |
| Tb.Th (mm) | 0.10 ± 0.01^ab^ | 0.10 ± 0.01^a^ | 0.09 ± 0.01^b^ | 0.09 ± 0.01^ab^ |
| Tb.Sp (mm) | 0.44 ± 0.10^ab^ | 0.55 ± 0.10^a^ | 0.37 ± 0.10^b^ | 0.55 ± 0.10^a^ |
| Tb.N (1/mm) | 1.50 ± 0.37^ab^ | 1.49 ± 0.41^ab^ | 1.76 ± 0.39^a^ | 1.14 ± 0.34^b^ |
